# Oophoropexy for Recurrent Ovarian Torsion

**DOI:** 10.1155/2018/8784958

**Published:** 2018-02-06

**Authors:** Jennifer Hartley, Muhammad Akhtar, Edmond Edi-Osagie

**Affiliations:** Saint Mary's Hospital, Central Manchester University Hospitals NHS Foundation Trust, Oxford Road, Manchester M13 9WL, UK

## Abstract

A 31-year-old nulliparous patient presents with a three-day history of right sided colicky abdominal pain and associated nausea. This patient has previously presented twice with right sided ovarian torsion with the background of polycystic ovaries in the last two consecutive years. Blood tests were normal. Due to previous history, there was a high index of clinical suspicion that this may be a further torsion. Therefore, the patient was taken to theatre for a diagnostic laparoscopy and a further right sided ovarian torsion was noted. At this time, oophoropexy was performed to the uterosacral ligament to prevent further torsion in order to preserve the patients' fertility. In this article, we detail this case and also provide a discussion of ovarian torsion including risk factors, presentation, and current thoughts on management.

## 1. Introduction

Ovarian torsion is a common gynaecological emergency with the majority of cases occurring in women of reproductive age [1]. Ovarian torsion involves the rotation of the ovary on its ligamentous supports often leading to interruption of its blood supply and in some cases necrosis. Prompt diagnosis is paramount to conserve ovarian and tubal function. Torsion is more likely to occur on the right side–possibly due to the fact that the infundibulopelvic ligament is longer on the right and/or due to the presence of the sigmoid colon preventing torsion on the left [2]. Ovarian torsion is also more likely to occur in adnexa with increased weight or diameter [3]. There have been some case reports linking torsion with polycystic ovarian syndrome due to increased ovarian volume [4]. Some experts advise that oophoropexy should be performed in cases of ovarian torsion in childhood to prevent recurrence particularly when one ovary has been removed [5]. However, there are doubts about the routine use of oophoropexy due to the lack of long-term follow-up studies in regard to its effect on future fertility.

## 2. Case Presentation

A 31-year-old nulliparous woman known to have polycystic ovaries presents to the emergency department with right sided lower abdominal pain. The patient states that the pain feels “exactly the same as when she previously had an ovarian torsion” with a very high pain score. The patient had suffered from right sided ovarian torsion twice in the last two years and both times had undergone laparoscopic detorsion by different surgeons.

The patient was unable to tolerate ultrasound on the day of admission. Blood tests were unremarkable apart from a mildly raised C-reactive protein (CRP) of 19 mg/L. The differential diagnosis consisted of recurrent ovarian torsion, ectopic pregnancy, tuboovarian abscess, and appendicitis. Ectopic pregnancy was ruled out with a negative serum *β*-HCG on admission. Tuboovarian abscess was felt unlikely to be the cause as the patient was apyrexial and inflammatory markers were normal. Appendicitis was also unlikely with normal inflammatory markers and no signs of peritonism. However, appendicitis would have been identifiable on the laparoscopy that was planned. Repeat ovarian torsion was the most likely diagnosis given her history and similar symptoms during her previous two admissions.

**Figure 1:**
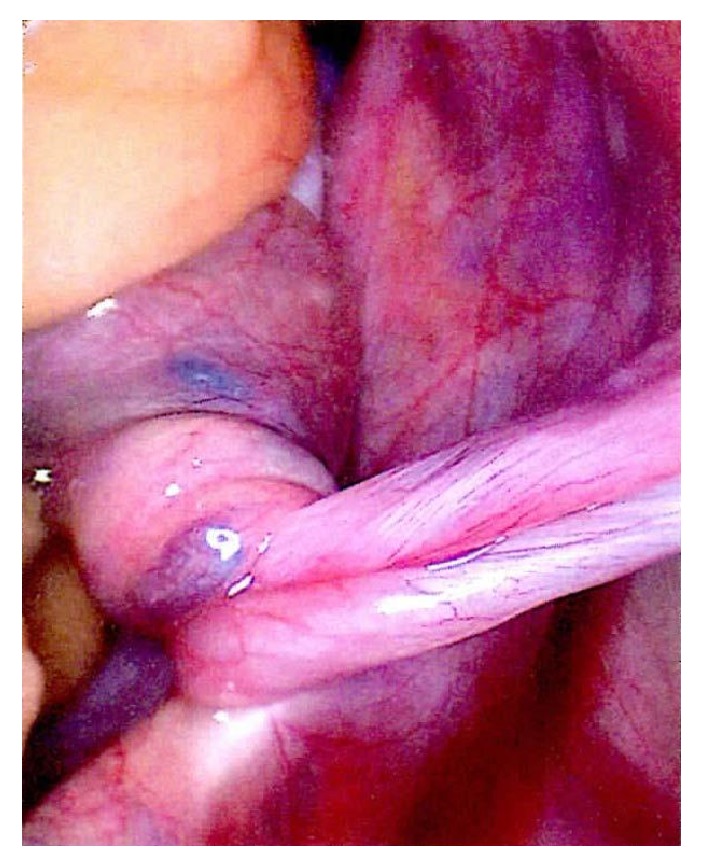
1st admission.

**Figure 2:**
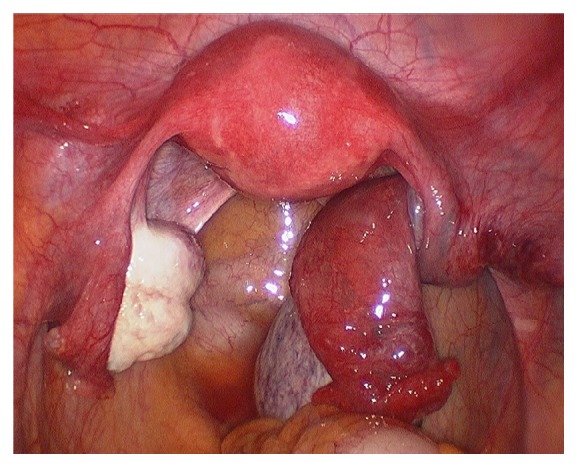
2nd admission.

**Figure 3:**
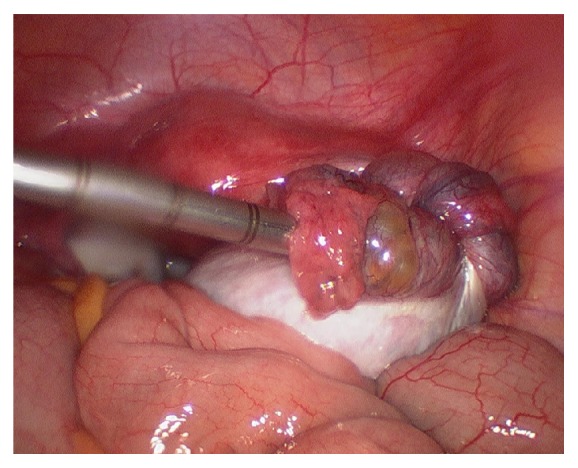
3rd admission.

**Figure 4:**
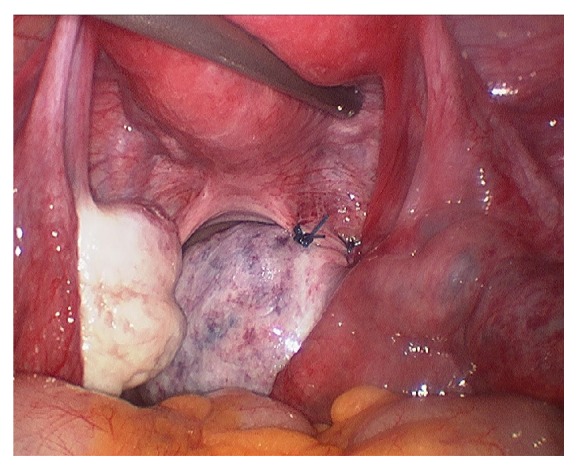
Fixation.

After informed consent, the patient underwent a diagnostic laparoscopy which revealed that the right ovary and tube had torted twice. The ovary and tube were untwisted (detorsion) and the right ovary was fixed to the right uterosacral ligament using two 1.0 PDS sutures (oophoropexy), as this was the third time she had suffered an ovarian torsion on the right side. The left tube and ovary were normal and the uterus was normal. The appendix was normal. In this case, this method of oophoropexy was chosen due to the absence of any notable uteroovarian ligament elongation and technical ease. Furthermore, by identifying the ureteric path prior to fixation of the ovary to the uterosacral ligament, we were able to avoid the pelvic side wall and therefore minimise the risk of damage to the ureter and blood vessels (see Figures 1, 2, 3, and 4).

The woman had unremarkable postoperative recovery. She had a pelvic ultrasound five weeks after operation, which showed both ovaries to be normal in size, shape, and echotexture with vascularity demonstrated within both ovaries using colour Doppler. The right ovary measured 34 × 14 × 32 mm.

## 3. Discussion

Adnexal torsion involves the rotation of the ovary on its ligamentous supports often leading to interruption of its blood supply and in some cases necrosis. Adnexal torsion accounts for 2.5–5% of all gynaecological emergencies [5]. Adnexal torsion is rare but its frequency is increasing with the increasing use of fertility treatments which can cause ovarian hyperstimulation. A high index of suspicion and subsequent rapid organisation of an emergency laparoscopy would ensure protection of future ovarian function and fertility.

Maintenance of fertility by acting rapidly when adnexal torsion is suspected is of paramount importance when considering that 70–80% of cases are encountered in women of reproductive age. There is an estimated pregnancy coexistence rate of 15–25% [1, 6, 7].

Rarely, delay or misdiagnosis may be responsible for potential fatal thrombophlebitis or peritonitis [3].

Torsion is more likely to occur on the right side, possibly due to the fact that the infundibulopelvic ligament is longer on the right and/or due to the presence of the sigmoid colon preventing torsion on the left [8].

Adnexal torsion is more likely to occur in ovaries with increased ovarian diameter or weight or in those with elongated infundibulopelvic ligaments [9].

Benign ovarian cysts other than endometriomas are more often the cause of torsion than neoplasms. This is thought to be because neoplasms along with endometriomas are often the source of adhesions or invade neighbouring tissues [3].

Benign cystic teratomas are more prone to torsion due to the increased weight and density of these cysts. Similarly polycystic are recognised to have tendencies for adnexal torsion. Tsafrir et al. (2012) reported polycystic ovaries to be present in 7% of 216 cases of torsion.

Torsion in pregnancy can be attributed to the additional weight of the corpus luteum. The corpus luteum produces progesterone required for continuing pregnancy during the first trimester before the placenta takes over production at twelve weeks. Therefore, it is recommended that progesterone is substituted in those women who undergo cystectomy or oophorectomy in the first trimester [8].

The symptoms of adnexal torsion include colicky abdominal pain, nausea, and vomiting. Pain lasting more than ten hours before surgery is associated with an increased rate of adnexal necrosis.

Patients with ovarian torsion can be febrile, particularly in cases of tissue necrosis. Torsion is challenging to diagnose; interestingly some reports have shown that half of all patients with torsion have suffered similar episodes of abdominal pain in the past. This knowledge is useful to consider when attempting differentiating torsion from appendicitis [9].

Doppler sonography remains the most useful investigation as reduced or missing flow in the ovary can provide evidence of torsion. However, Peña et al. (2000) found that 60% of cases of torsion are missed by Doppler, although its positive predictive value is 100% [10]. Doppler sonography is limited in the fact that it can only diagnose interruption of arterial flow. It cannot diagnose interferences in venous flow which can often precede arterial interruptions.

Laparoscopy is the gold standard for diagnosis of adnexal torsion [8].

Laparoscopy in pregnancy has divided opinion. Nezhat et al. (1997) demonstrate the benefits of operative laparoscopy and subsequent successful pregnancy outcomes [11]. Schelling (2000) recommends laparoscopy to be avoided in pregnancy due to complicated access, prolonged operating times, and a theoretical risk of fetal acidosis due to an increased abdominal pressure and consequent decrease in uterine perfusion [12].

Pucci and Seed (1991) report that no adverse fetal effects due to carbon dioxide pneumoperitoneum have been detected [15]. Taking this into consideration, when operating on pregnant women certain steps are recommended. These include monitoring arterial blood gas and carbon dioxide level and avoiding pneumoperitoneum pressure greater than 12 mmHg and left lateral positioning.

Conservative treatment consists of untwisting the adnexa (detorsion). More recently, debate has sparked over the issue of oophoropexy and what the best practice is. Oophoropexy or fixation of the ovary is performed with the aim of reducing the risk of further episodes of torsion, therefore maintaining long-term fertility. However, there is a lack of evidence regarding the long-term outcome of oophoropexy. Theoretical concerns in regard to oophoropexy include concerns over the interference of tubal blood supply or a disruption to the communication between ovary and fallopian tube [16].

There is also debate over the appropriateness of oophoropexy of the contralateral ovary in cases of children who have lost an ovary due to torsion and necrosis. Once a child has lost one ovary they are at risk of asynchronous torsion of the contralateral ovary which can be catastrophic for that child's future reproductive health [9].

Methods of oophoropexy include fixation of the ovary to the pelvic side wall, posterior abdominal wall, or the posterior wall of the uterus. Plication of the uteroovarian ligaments is another method which can be employed to prevent recurrence. On review of the literature, plication of the uteroovarian ligament is the preferred technique of oophoropexy as it supposedly has minimal effect on fertility outcome. A combined approach of fixation of the ovary and shortening of the ligament may be more efficacious in preventing recurrence [5] (see Table 1).

One case report details a patient who suffered six separate episodes of torsion and two failed oophoropexies to the pelvic side wall. This patient subsequently underwent elective uteroovarian ligament shortening with no further episodes. This brings to light the issue of timing when performing oophoropexy due to the potential issue of suture instability in fragile, oedematous, and/or ischaemic tissue when oophoropexy is performed at the time of diagnosis of torsion [5].

## 4. Conclusion

In conclusion, it is evident that studies comparing long-term results of various methods of oophoropexy are needed. Evidence is also lacking in terms of the preferable timing of oophoropexy and whether the contralateral adnexa should also be fixed. It is also clear that emergency laparoscopy is the only reliable way to detect an adnexal torsion. Proceeding to laparoscopy should be prompt in order to prevent any complications which could have a detrimental effect on the patients' future fertility. In the meantime, a case by case approach should be employed with attention to potential risks of recurrence such as adnexal masses, polycystic ovaries, and the presence of large ovarian cysts.

Oophoropexy is an effective surgical method to prevent recurrence after two or more episodes of ovarian torsion; plication of uteroovarian ligaments remains the most anatomically feasible method.

The efficacy and safety of oophoropexy are not well established.

Evidence is based upon small case series and anecdotal case reports of different approaches of oophoropexy.

The impact of oophoropexy on subsequent fertility and its efficacy to prevent future recurrences merit further study.

## Figures and Tables

**Table 1:** 

Surgical techniques for oophoropexy
Fixation of the ovary to posterior abdominal wall [13]
Fixation of the ovary to pelvic side wall [8]
Plication of the uteroovarian ligaments [14]
Uteroovarian ligament shortening by “endoloop application” [4]
Combined approach of fixation of the ovary and shortening of the ligament [5]

## Additional Points

This case occurred at Saint Mary's Hospital, Manchester. The patient was under the care of Mr. Edi-Osagie, Consultant Gynaecologist. Both Dr. Hartley and Dr. Akhtar were trainees involved in the management. This case is relevant as it is unusual to see a case of recurrent torsion three times in the same patient. Our review demonstrates that we lack evidence on the best approach to take in regard to recurrent ovarian torsion.
